# Bis(1,10-phenanthroline-5,6-dione-κ^2^ *N*,*N*′)silver(I) tetra­fluoridoborate

**DOI:** 10.1107/S160053680903222X

**Published:** 2009-08-22

**Authors:** Jonathan Onuegbu, Ray J. Butcher, Charles Hosten, Uche Charles Udeochu, Oladapo Bakare

**Affiliations:** a Department of Chemistry, Howard University, 525 College Street NW, Washington, DC 20059, USA

## Abstract

In the structure of the title compound, [Ag(C_12_H_6_N_2_O_2_)_2_]BF_4_ or [Ag*L*_2_]BF_4_ (*L* = phendione), the Ag and B atoms are located on twofold rotation axes. The dihedral angle between the two phendione ligands is 36.7 (2)°. The coordination about the Ag^I^ center is distorted tetra­hedral (τ_4_ = 0.546). The crystal structure is consolidated by weak C—H⋯O(phendione) and C—H⋯F(BF_4_^−^) inter­actions. The BF_4_^−^ counter-anion is strongly disordered and was modelled with two sets of idealized F atoms.

## Related literature

For the different coordination properties of phendione, see: Calderazzo *et al.* (1999 ▶, 2002 ▶); Calucci *et al.* (2006 ▶); Galet *et al.* (2005 ▶); Lei *et al.* (1996 ▶); Okamura *et al.* (2006 ▶). For examples with phendione ligands where N and O donors are used simultaneously, see: Fox *et al.* (1991 ▶); Shavaleev *et al.* (2003**a* ▶,b* ▶); Ruiz *et al.* (1999 ▶); Paw & Eisenberg (1997 ▶). Similar structures containing Ag have also been reported by Onuegbu *et al.* (2007 ▶). For background to phendione chemistry, see: Udeochu *et al.* (2007 ▶); Onuegbu *et al.* (2007 ▶). For reference structural data, see: Allen (2002 ▶); Leschke *et al.* (2002 ▶); Paramonov *et al.* (2003 ▶); Pallenberg *et al.* (1997 ▶); Titze *et al.* (1997 ▶). Details of the τ_4_ parameter were given by Yang *et al.* (2007 ▶).
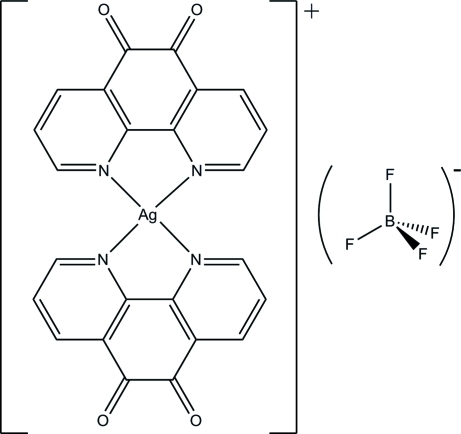


## Experimental

### 

#### Crystal data

[Ag(C_12_H_6_N_2_O_2_)_2_]BF_4_
*M* *_r_* = 615.06
Monoclinic, 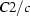

*a* = 13.2249 (6) Å
*b* = 12.0115 (17) Å
*c* = 14.4338 (7) Å
β = 108.481 (5)°
*V* = 2174.6 (3) Å^3^
*Z* = 4
Mo *K*α radiation
μ = 1.01 mm^−1^
*T* = 200 K
0.44 × 0.37 × 0.28 mm

#### Data collection

Oxford Diffraction Gemini R diffractometer
Absorption correction: multi-scan (*CrysAlis RED*; Oxford Diffraction, 2007 ▶) *T*_min_ = 0.856, *T*_max_ = 1.000 (expected range = 0.646–0.755)
11815 measured reflections
3647 independent reflections
2306 reflections with *I* > 2σ(*I*)
*R*_int_ = 0.024

#### Refinement

*R*[*F* ^2^ > 2σ(*F* ^2^)] = 0.047
*wR*(*F* ^2^) = 0.134
*S* = 0.98
3647 reflections
204 parameters
32 restraints
H-atom parameters constrained
Δρ_max_ = 1.81 e Å^−3^
Δρ_min_ = −1.39 e Å^−3^

### 

Data collection: *CrysAlis CCD* (Oxford Diffraction, 2007 ▶); cell refinement: *CrysAlis RED* (Oxford Diffraction, 2007 ▶); data reduction: *CrysAlis RED*; program(s) used to solve structure: *SHELXS97* (Sheldrick, 2008 ▶); program(s) used to refine structure: *SHELXL97* (Sheldrick, 2008 ▶); molecular graphics: *SHELXTL* (Sheldrick, 2008 ▶); software used to prepare material for publication: *SHELXTL*.

## Supplementary Material

Crystal structure: contains datablocks global, I. DOI: 10.1107/S160053680903222X/wm2249sup1.cif

Structure factors: contains datablocks I. DOI: 10.1107/S160053680903222X/wm2249Isup2.hkl

Additional supplementary materials:  crystallographic information; 3D view; checkCIF report

## Figures and Tables

**Table 1:** Selected bond lengths (Å)

Ag—N1	2.356 (2)
Ag—N2	2.357 (2)

**Table 2:** Hydrogen-bond geometry (Å, °)

*D*—H⋯*A*	*D*—H	H⋯*A*	*D*⋯*A*	*D*—H⋯*A*
C3—H3*A*⋯O1^i^	0.95	2.51	3.347 (4)	147
C1—H1*A*⋯F1*A*^ii^	0.95	2.35	3.083 (6)	133
C2—H2*A*⋯F1*B*	0.95	2.17	2.803 (8)	123
C10—H10*A*⋯F2*A*^iii^	0.95	2.24	2.859 (5)	122
C10—H10*A*⋯F2*B*^iv^	0.95	2.28	3.065 (4)	140

Symmetry codes: (i) 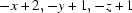; (ii) 
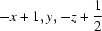
; (iii) 
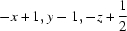
; (iv) 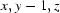.

**Fig. 1.**
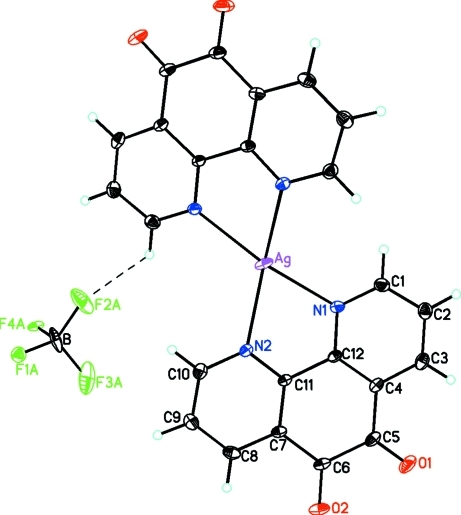
View of (I), [Ag(L)2]+(BF4)-, showing the atom-labelling scheme. The C—H···F interaction is shown as a dashed line. Unlabeled atoms are related by -x, y, 1/2 - z. Displacement ellipsoids are drawn at the 20% probability level. Only the major component of the disordered anion is shown.

**Fig. 2.**
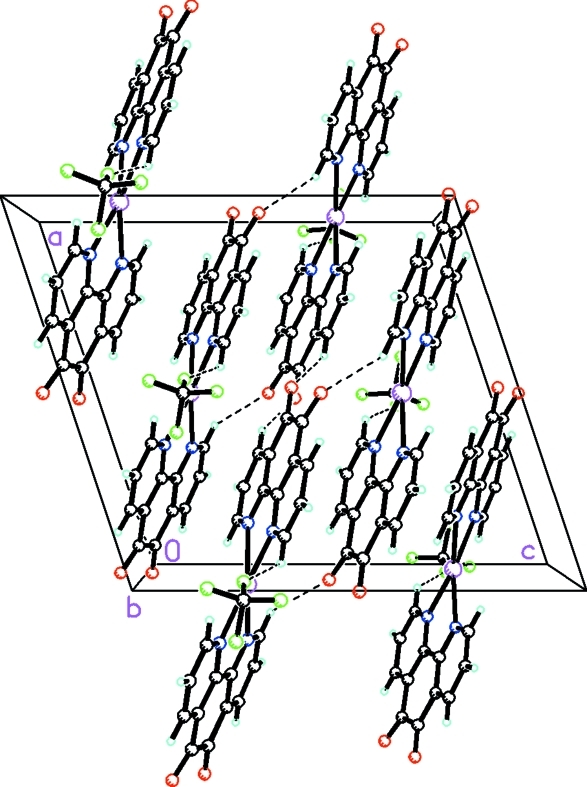
The molecular packing of (I) viewed approximately along the b axis. Dotted lines indicate the C—H···O and C—H···F interactions. Only the major component of the disordered anion is shown.

## supplementary crystallographic information

### Comment

Phendione (1,10-phenanthroline-5,6-dione) is an excellent ligand that incorporates two functional groups with different coordination properties (Calderazzo *et al.*, 1999, 2002; Calucci *et al.*, 2006; Galet *et al.*, 2005; Lei *et al.*, 1996; Okamura *et al.*, 2006). This well known ligand possesses both the α-diimine and orthoquinone moieties. While phendione usually binds to metals through its imine N atoms, in some cases both the N and O donors are used simultaneously (Fox *et al.*, 1991; Shavaleev *et al.*, 2003*a, b*; Ruiz *et al.*, 1999; Paw & Eisenberg, 1997). The crystal structures of two complexes of copper and phendione have been determined (Galet *et al.*, 2005;). Similar structures containing Ag have also been reported (Onuegbu *et al.*, 2007). In this paper, as part of our study of phendione chemistry (Udeochu *et al.*, 2007; Onuegbu *et al.*, 2007), we report the synthesis and characterization of the title compound, [Ag*L*_2_]^+^(BF_4_)^-^, (I).

The structure of (I), shown in Figure 1, is made up of an [Ag(*L*)_2_]^+^ cation and a tetrafluoridoborate anion. The silver atom is coordinated to the two nitrogen atoms of both phendione ligands. Both the Ag atom of the cation and the B atom of the anion lie on a crystallographic twofold rotation axes. The C═O bond lengths in the phendione ligands (1.210 (4) and 1.206 (3) Å) are comparable to those values found in other such complexes (Allen, 2002; Onuegbu *et al.*, 2007). The Ag—N bond lengths (2.356 (2) and 2.357 (2) Å) are similar to those found in related phenanthroline derivatives of silver (Leschke *et al.*, 2002; Paramonov *et al.*, 2003; Pallenberg *et al.*, 1997; Titze *et al.*, 1997).

In (I), the silver cation is in a distorted tetrahedral environment. This is best illustrated by the dihedral angle between the planes of the coordinated ligands which in this case the angle is 36.7 (2)°. This compares with values of 36.8 (2)° found in the analogous perchlorate analog and the values of 32.4° and 70.5° found for other structurally characterized Ag complexes containing the bis(1,10-phenanthroline) core. Another recent parameter for 4-coordinate complexes (τ_4_, Yang *et al.*, 2007) has been developed to place a structure on the continuum between square planar (0) and tetrahedral (1). For the present structure this value is 0.546.

Copper forms a similar complex with phendione. However, in this case the twofold axis passes between the phendione ligands with a dihedral angle of 44.5° between them.

In the structure of (I), there are weak C—H···O(phendione) and C—H···F(BF_4_^-^) interactions (Fig. 2).

### Experimental

A flask containing 1,10-phenanthroline hydrate (1.00 g, 5.04 mmol) and potassium bromide (5.95 g, 50.0 mmol) was placed in an ice bath. Concentrated sulfuric acid (20 cm^3^) was added in small portions, followed by drop-wise addition of concentrated nitric acid (10 cm^3^). The resulting solution was heated for 2 h at 253–257 K and cooled to room temperature. The solution was then poured into 400 cm^3^ of water and neutralized with sodium bicarbonate, after which the phendione was extracted with dichloromethane, and recrystallized using a methanol-water mixture.

The title compound was synthesized in an atmosphere saturated with N_2_. To a solution of tetrakis(acetonitrile)silver(I)tetrafluoridoborate (0.0843 g) in 10 ml of CH_3_CN, was added drop-wise a solution (10 ml) of CH_3_CN containing 0.0492 g of phendione. The final yellowish solution was filtered and allowed to slowly evaporate yielding reddish brown crystals of the title compound suitable for X-ray studies.

### Refinement

H atoms were placed in geometrically idealized positions and constrained to ride on their parent atoms with C—H distances of 0.95 Å and *U*_iso_(H) = 1.2*U*_eq_(C). The tetrafluoridoborate anion is disordered. Two sets of F atoms, constrained to meet the criteria for idealized tetrahedra, were used with occupancy factors of 0.406 (4) and 0.096 (4). The temperature factors for the major component were refined anisotropically and constrained by use of the SIMU and DELU instructions in *SHELXL97* (Sheldrick, 2008). In the final difference Fourier there were positive and negative holes of +1.807 and -1.393 eA^-3^ near the disordered F atoms.

### Crystal data

**Table:**

[Ag(C_12_H_6_N_2_O_2_)_2_]BF_4_	*F*(000) = 1216
*M**_r_* = 615.06	*D*_x_ = 1.879 Mg m^−^^3^
Monoclinic, *C*2/*c*	Mo *K*α radiation, λ = 0.71073 Å
Hall symbol: -C 2yc	Cell parameters from 5444 reflections
*a* = 13.2249 (6) Å	θ = 4.6–32.4°
*b* = 12.0115 (17) Å	µ = 1.00 mm^−^^1^
*c* = 14.4338 (7) Å	*T* = 200 K
β = 108.481 (5)°	Prism, colorless
*V* = 2174.6 (3) Å^3^	0.44 × 0.37 × 0.28 mm
*Z* = 4	

### Data collection

**Table:**

Oxford Diffraction Gemini R diffractometer	3647 independent reflections
Radiation source: fine-focus sealed tube	2306 reflections with *I* > 2σ(*I*)
graphite	*R*_int_ = 0.024
Detector resolution: 10.5081 pixels mm^-1^	θ_max_ = 32.5°, θ_min_ = 4.6°
φ and ω scans	*h* = −19→19
Absorption correction: multi-scan (*CrysAlis RED*; Oxford Diffraction, 2007)	*k* = −17→17
*T*_min_ = 0.856, *T*_max_ = 1.000	*l* = −16→21
11815 measured reflections	

### Refinement

**Table:**

Refinement on *F*^2^	Primary atom site location: structure-invariant direct methods
Least-squares matrix: full	Secondary atom site location: difference Fourier map
*R*[*F*^2^ > 2σ(*F*^2^)] = 0.047	Hydrogen site location: inferred from neighbouring sites
*wR*(*F*^2^) = 0.134	H-atom parameters constrained
*S* = 0.98	*w* = 1/[σ^2^(*F*_o_^2^) + (0.087*P*)^2^] where *P* = (*F*_o_^2^ + 2*F*_c_^2^)/3
3647 reflections	(Δ/σ)_max_ < 0.001
204 parameters	Δρ_max_ = 1.81 e Å^−^^3^
32 restraints	Δρ_min_ = −1.39 e Å^−^^3^

### Special details

**Table:**

Geometry. All e.s.d.'s (except the e.s.d. in the dihedral angle between two l.s. planes) are estimated using the full covariance matrix. The cell e.s.d.'s are taken into account individually in the estimation of e.s.d.'s in distances, angles and torsion angles; correlations between e.s.d.'s in cell parameters are only used when they are defined by crystal symmetry. An approximate (isotropic) treatment of cell e.s.d.'s is used for estimating e.s.d.'s involving l.s. planes.
Refinement. Refinement of *F*^2^ against ALL reflections. The weighted *R*-factor *wR* and goodness of fit *S* are based on *F*^2^, conventional *R*-factors *R* are based on *F*, with *F* set to zero for negative *F*^2^. The threshold expression of *F*^2^ > σ(*F*^2^) is used only for calculating *R*-factors(gt) *etc*. and is not relevant to the choice of reflections for refinement. *R*-factors based on *F*^2^ are statistically about twice as large as those based on *F*, and *R*- factors based on ALL data will be even larger.

### Fractional atomic coordinates and isotropic or equivalent isotropic displacement parameters (Å^2^)

**Table:**

	*x*	*y*	*z*	*U*_iso_*/*U*_eq_	Occ. (<1)
Ag	0.5000	0.21159 (3)	0.2500	0.04327 (15)	
O1	1.01490 (18)	0.3415 (2)	0.5002 (2)	0.0563 (7)	
O2	0.99985 (17)	0.1301 (2)	0.56180 (17)	0.0507 (6)	
N1	0.65530 (18)	0.3220 (2)	0.29553 (17)	0.0304 (5)	
N2	0.64367 (17)	0.10772 (19)	0.35180 (17)	0.0284 (5)	
C1	0.6565 (2)	0.4291 (3)	0.2713 (2)	0.0371 (6)	
H1A	0.5941	0.4599	0.2261	0.044*	
C2	0.7443 (2)	0.4976 (3)	0.3086 (2)	0.0413 (7)	
H2A	0.7414	0.5742	0.2914	0.050*	
C3	0.8357 (2)	0.4515 (3)	0.3713 (2)	0.0387 (7)	
H3A	0.8980	0.4957	0.3966	0.046*	
C4	0.8364 (2)	0.3402 (2)	0.39734 (19)	0.0294 (5)	
C5	0.9324 (2)	0.2908 (2)	0.4662 (2)	0.0356 (6)	
C6	0.9259 (2)	0.1693 (3)	0.4981 (2)	0.0329 (6)	
C7	0.82541 (19)	0.1076 (2)	0.45309 (18)	0.0282 (5)	
C8	0.8176 (2)	−0.0024 (3)	0.4776 (2)	0.0373 (7)	
H8A	0.8766	−0.0393	0.5224	0.045*	
C9	0.7227 (2)	−0.0579 (3)	0.4360 (2)	0.0404 (7)	
H9A	0.7159	−0.1346	0.4489	0.048*	
C10	0.6377 (2)	0.0010 (2)	0.3753 (2)	0.0370 (6)	
H10A	0.5716	−0.0365	0.3489	0.044*	
C12	0.7426 (2)	0.2784 (2)	0.35847 (19)	0.0255 (5)	
C11	0.73725 (19)	0.1602 (2)	0.38786 (18)	0.0249 (5)	
B	0.5000	0.7040 (2)	0.2500	0.060 (2)	
F1A	0.4655 (3)	0.6438 (4)	0.3134 (3)	0.0308 (13)	0.406 (4)
F2A	0.4411 (3)	0.7977 (3)	0.2246 (4)	0.107 (3)	0.406 (4)
F3A	0.60395 (19)	0.7324 (4)	0.2940 (3)	0.113 (3)	0.406 (4)
F4A	0.4924 (4)	0.6425 (5)	0.1696 (3)	0.0439 (18)	0.406 (4)
F1B	0.5925 (6)	0.6663 (3)	0.2398 (12)	0.053 (4)*	0.094 (4)
F2B	0.5000	0.8168 (3)	0.2500	0.053 (4)*	0.189 (8)
F3B	0.4168 (8)	0.6665 (3)	0.1746 (7)	0.053 (4)*	0.094 (4)
F4B	0.4905 (13)	0.6663 (3)	0.3353 (5)	0.053 (4)*	0.094 (4)

### Atomic displacement parameters (Å^2^)

**Table:**

	*U*^11^	*U*^22^	*U*^33^	*U*^12^	*U*^13^	*U*^23^
Ag	0.02050 (16)	0.0611 (3)	0.0386 (2)	0.000	−0.00431 (12)	0.000
O1	0.0270 (11)	0.0649 (16)	0.0674 (17)	−0.0135 (11)	0.0012 (11)	−0.0144 (14)
O2	0.0269 (10)	0.0765 (16)	0.0400 (12)	0.0105 (10)	−0.0016 (9)	0.0064 (12)
N1	0.0259 (11)	0.0366 (12)	0.0260 (11)	0.0005 (9)	0.0045 (9)	0.0018 (9)
N2	0.0236 (10)	0.0309 (12)	0.0281 (11)	−0.0015 (8)	0.0043 (8)	−0.0018 (9)
C1	0.0384 (14)	0.0394 (16)	0.0301 (14)	0.0047 (12)	0.0061 (12)	0.0040 (12)
C2	0.0499 (17)	0.0374 (16)	0.0387 (17)	−0.0061 (14)	0.0171 (14)	0.0010 (13)
C3	0.0368 (14)	0.0429 (17)	0.0407 (16)	−0.0119 (12)	0.0185 (13)	−0.0085 (13)
C4	0.0255 (12)	0.0387 (15)	0.0257 (12)	−0.0073 (11)	0.0104 (10)	−0.0074 (11)
C5	0.0234 (12)	0.0477 (17)	0.0352 (15)	−0.0040 (11)	0.0087 (11)	−0.0133 (13)
C6	0.0216 (11)	0.0519 (16)	0.0228 (12)	0.0036 (11)	0.0037 (9)	−0.0026 (12)
C7	0.0212 (11)	0.0392 (15)	0.0235 (12)	0.0050 (10)	0.0062 (9)	−0.0006 (10)
C8	0.0347 (14)	0.0453 (17)	0.0332 (15)	0.0138 (12)	0.0126 (12)	0.0056 (12)
C9	0.0442 (16)	0.0338 (15)	0.0457 (18)	0.0038 (13)	0.0180 (14)	0.0061 (13)
C10	0.0335 (13)	0.0398 (16)	0.0400 (16)	−0.0065 (12)	0.0150 (12)	−0.0032 (12)
C12	0.0219 (11)	0.0349 (14)	0.0197 (11)	−0.0028 (9)	0.0067 (9)	−0.0035 (10)
C11	0.0207 (10)	0.0310 (13)	0.0218 (11)	0.0015 (10)	0.0048 (8)	−0.0027 (10)
B	0.110 (6)	0.009 (2)	0.085 (5)	0.000	0.065 (5)	0.000
F1A	0.032 (3)	0.036 (3)	0.022 (2)	−0.022 (2)	0.006 (2)	−0.0135 (18)
F2A	0.189 (9)	0.027 (3)	0.124 (7)	0.049 (3)	0.077 (6)	0.031 (3)
F3A	0.141 (7)	0.044 (4)	0.170 (8)	−0.033 (4)	0.071 (6)	−0.051 (5)
F4A	0.046 (4)	0.051 (3)	0.027 (3)	0.023 (3)	0.001 (2)	0.003 (2)

### Geometric parameters (Å, °)

**Table:**

Ag—N1	2.356 (2)	C5—C6	1.541 (5)
Ag—N1^i^	2.356 (2)	C6—C7	1.480 (4)
Ag—N2^i^	2.357 (2)	C7—C8	1.381 (4)
Ag—N2	2.357 (2)	C7—C11	1.396 (3)
O1—C5	1.210 (4)	C8—C9	1.379 (4)
O2—C6	1.206 (3)	C8—H8A	0.9500
N1—C12	1.329 (3)	C9—C10	1.380 (4)
N1—C1	1.335 (4)	C9—H9A	0.9500
N2—C10	1.335 (4)	C10—H10A	0.9500
N2—C11	1.338 (3)	C12—C11	1.489 (4)
C1—C2	1.385 (4)	B—F1A	1.354 (2)
C1—H1A	0.9500	B—F2A	1.352 (2)
C2—C3	1.377 (4)	B—F3A	1.362 (2)
C2—H2A	0.9500	B—F4A	1.352 (2)
C3—C4	1.389 (4)	B—F1B	1.355 (2)
C3—H3A	0.9500	B—F2B	1.355 (2)
C4—C12	1.402 (4)	B—F3B	1.356 (2)
C4—C5	1.466 (4)	B—F4B	1.355 (2)
			
N1—Ag—N1^i^	111.52 (12)	C8—C7—C11	119.5 (2)
N1—Ag—N2^i^	158.56 (8)	C8—C7—C6	119.8 (3)
N1^i^—Ag—N2^i^	70.42 (8)	C11—C7—C6	120.7 (2)
N1—Ag—N2	70.42 (8)	C9—C8—C7	118.8 (3)
N1^i^—Ag—N2	158.56 (8)	C9—C8—H8A	120.6
N2^i^—Ag—N2	116.08 (11)	C7—C8—H8A	120.6
C12—N1—C1	118.6 (2)	C8—C9—C10	118.3 (3)
C12—N1—Ag	117.40 (18)	C8—C9—H9A	120.8
C1—N1—Ag	123.46 (19)	C10—C9—H9A	120.8
C10—N2—C11	118.4 (2)	N2—C10—C9	123.5 (3)
C10—N2—Ag	124.49 (19)	N2—C10—H10A	118.3
C11—N2—Ag	117.15 (17)	C9—C10—H10A	118.3
N1—C1—C2	123.3 (3)	N1—C12—C4	122.1 (2)
N1—C1—H1A	118.3	N1—C12—C11	117.4 (2)
C2—C1—H1A	118.3	C4—C12—C11	120.4 (2)
C3—C2—C1	118.0 (3)	N2—C11—C7	121.4 (2)
C3—C2—H2A	121.0	N2—C11—C12	117.4 (2)
C1—C2—H2A	121.0	C7—C11—C12	121.2 (2)
C2—C3—C4	119.6 (3)	F2A—B—F4A	110.07 (10)
C2—C3—H3A	120.2	F2A—B—F1A	109.81 (10)
C4—C3—H3A	120.2	F4A—B—F1A	109.81 (9)
C3—C4—C12	118.2 (3)	F1A—B—F1B	113.8 (5)
C3—C4—C5	120.2 (2)	F2B—B—F1B	109.52 (10)
C12—C4—C5	121.6 (3)	F2A—B—F4B	108.2 (5)
O1—C5—C4	123.2 (3)	F2B—B—F4B	109.52 (10)
O1—C5—C6	119.0 (3)	F1B—B—F4B	109.52 (10)
C4—C5—C6	117.9 (2)	F4A^i^—B—F3B	109.1 (6)
O2—C6—C7	122.5 (3)	F2B—B—F3B	109.43 (10)
O2—C6—C5	119.3 (3)	F1B—B—F3B	109.43 (10)
C7—C6—C5	118.1 (2)	F4B—B—F3B	109.41 (10)
			
N1^i^—Ag—N1—C12	152.8 (2)	O2—C6—C7—C11	172.5 (3)
N2^i^—Ag—N1—C12	−116.1 (2)	C5—C6—C7—C11	−3.7 (4)
N2—Ag—N1—C12	−4.37 (18)	C11—C7—C8—C9	0.6 (4)
N1^i^—Ag—N1—C1	−18.6 (2)	C6—C7—C8—C9	178.9 (3)
N2^i^—Ag—N1—C1	72.5 (3)	C7—C8—C9—C10	−3.2 (4)
N2—Ag—N1—C1	−175.8 (2)	C11—N2—C10—C9	1.0 (4)
N1—Ag—N2—C10	−176.9 (3)	Ag—N2—C10—C9	−179.2 (2)
N1^i^—Ag—N2—C10	83.9 (3)	C8—C9—C10—N2	2.5 (5)
N2^i^—Ag—N2—C10	−19.1 (2)	C1—N1—C12—C4	−2.2 (4)
N1—Ag—N2—C11	2.92 (18)	Ag—N1—C12—C4	−174.04 (19)
N1^i^—Ag—N2—C11	−96.3 (3)	C1—N1—C12—C11	177.1 (2)
N2^i^—Ag—N2—C11	160.7 (2)	Ag—N1—C12—C11	5.3 (3)
C12—N1—C1—C2	−0.2 (4)	C3—C4—C12—N1	2.4 (4)
Ag—N1—C1—C2	171.1 (2)	C5—C4—C12—N1	−179.6 (2)
N1—C1—C2—C3	2.2 (5)	C3—C4—C12—C11	−176.9 (2)
C1—C2—C3—C4	−2.0 (4)	C5—C4—C12—C11	1.1 (4)
C2—C3—C4—C12	−0.2 (4)	C10—N2—C11—C7	−3.7 (4)
C2—C3—C4—C5	−178.3 (3)	Ag—N2—C11—C7	176.42 (18)
C3—C4—C5—O1	−2.8 (5)	C10—N2—C11—C12	178.4 (2)
C12—C4—C5—O1	179.2 (3)	Ag—N2—C11—C12	−1.4 (3)
C3—C4—C5—C6	175.6 (3)	C8—C7—C11—N2	3.0 (4)
C12—C4—C5—C6	−2.4 (4)	C6—C7—C11—N2	−175.3 (2)
O1—C5—C6—O2	5.7 (5)	C8—C7—C11—C12	−179.3 (2)
C4—C5—C6—O2	−172.7 (3)	C6—C7—C11—C12	2.4 (4)
O1—C5—C6—C7	−177.9 (3)	N1—C12—C11—N2	−2.6 (4)
C4—C5—C6—C7	3.6 (4)	C4—C12—C11—N2	176.7 (2)
O2—C6—C7—C8	−5.8 (4)	N1—C12—C11—C7	179.5 (2)
C5—C6—C7—C8	178.0 (3)	C4—C12—C11—C7	−1.1 (4)

Symmetry codes: (i) −*x*+1, *y*, −*z*+1/2.

### Hydrogen-bond geometry (Å, °)

**Table:**

*D*—H···*A*	*D*—H	H···*A*	*D*···*A*	*D*—H···*A*
C3—H3A···O1^ii^	0.95	2.51	3.347 (4)	147
C1—H1A···F1A^i^	0.95	2.35	3.083 (6)	133
C2—H2A···F1B	0.95	2.17	2.803 (8)	123
C10—H10A···F2A^iii^	0.95	2.24	2.859 (5)	122
C10—H10A···F2B^iv^	0.95	2.28	3.065 (4)	140

Symmetry codes: (ii) −*x*+2, −*y*+1, −*z*+1; (i) −*x*+1, *y*, −*z*+1/2; (iii) −*x*+1, *y*−1, −*z*+1/2; (iv) *x*, *y*−1, *z*.
